# Exceptional Aging: Cognitive and Brain Health in Super Movers

**DOI:** 10.1093/geroni/igaf122.4112

**Published:** 2025-12-31

**Authors:** Oshadi Jayakody, Joe Verghese, Helena Blumen, Sofiya Milman, Nir Barzilai, Ying Jin, Cuiling Wang, Erica Weiss

**Affiliations:** Albert Einstein College of Medicine, Bronx, New York, United States; Stony Brook University, Stony Brook, New York, United States; Stony Brook University, Stony Brook, New York, United States; Albert Einstein College of Medicine, Bronx, New York, United States; Institute for Aging Research, Bronx, New York, United States; Albert Einstein College of Medicine, Bronx, New York, United States; Albert Einstein College of Medicine, Bronx, New York, United States; Albert Einstein College of Medicine, Bronx, New York, United States

## Abstract

Super movers, individuals aged 80 and older with gait speeds ≥1.5 SD above age- and sex-adjusted means, represent an exceptional aging phenotype and may offer insights into resilience against cognitive decline. We examined their risk of incident cognitive impairment, cognitive trajectories and brain health using data from 1) five Health and Retirement Study International Network of Studies (HRS-INS) 2) the LonGenity study and 3) the RUSH Memory Aging Project (RUSH MAP). In HRS-INS we assessed incident cognitive impairment (>1.5 SD below age-adjusted cognitive test means plus impaired Instrumental Activities of Daily Living) of super movers and conduct a meta-analysis using age- and sex-adjusted hazard ratios (HR) from Cox models of individual studies. LonGenity study data were used to model longitudinal cognitive decline using linear mixed-effects models (adjusted for age, sex, education, and parental longevity) and to compare cortical thickness and hippocampal subfield volumes between super versus non-super movers. RUSH MAP data assessed dementia-related pathology in super movers. In pooled HRS-INS data (n = 358/3,989 super movers; baseline age 83.6–84.4 years; follow-up 3.8–6.1 years), super movers had a lower risk of cognitive impairment (HR 0.50, 95% CI 0.29–0.71). In LonGenity (n = 197; mean age 84.6, SD 3.3), super movers exhibited slower decline in memory and non-memory domains and preserved hippocampal subfield volumes. In RUSH MAP, they had better late-life cognition, despite no differences in dementia-related pathology. Understanding the behavioral and biological traits of super movers may reveal protective mechanisms against cognitive decline and dementia to inform future interventions.

